# Guidelines for Enhanced Recovery After Trauma and Intensive Care (ERATIC): Enhanced Recovery After Surgery (ERAS) and International Association for Trauma Surgery and Intensive Care (IATSIC) Society Recommendations: Part 3: Trauma Ethics and Systems Aspects

**DOI:** 10.1002/wjs.70003

**Published:** 2025-07-22

**Authors:** Timothy C. Hardcastle, Christine Gaarder, Zsolt Balogh, Scott D'amours, Kimberly A. Davis, Amit Gupta, Shahin Mohseni, Paal A. Naess, Shanisa Naidoo, Tarek Razek, Simon Robertson, Hayaki Uchino, David Zonies, Jade Whing, Michael J. Scott

**Affiliations:** ^1^ Inkosi Albert Luthuli Central Hospital Durban South Africa; ^2^ Department of Surgical Sciences University of KwaZulu‐Natal Durban South Africa; ^3^ Oslo University Hospital Ulleval and University of Oslo Oslo Norway; ^4^ John Hunter Hospital and University of Newcastle Newcastle Australia; ^5^ Liverpool Hospital and University of Sydney Sydney Australia; ^6^ Division of General Surgery Trauma and Surgical Critical Care Yale School of Medicine New Haven Connecticut USA; ^7^ JPN Apex Trauma Centre All India Institute of Medical Sciences New Delhi India; ^8^ Department of Surgery New Al Jahra Hospital Al Jahra Kuwait; ^9^ School of Medical Sciences Orebro University Orebro Sweden; ^10^ Montreal University Hospital and McGill University Montreal Canada; ^11^ Canberra Hospital Canberra Australia; ^12^ Department of Surgery University of Washington Seattle Washington USA; ^13^ Norfolk and Norwich University Hospital Norwich UK; ^14^ Department of Anesthesiology and Perioperative Medicine University of Rochester Rochester New York USA; ^15^ Leonard Davis Institute for Health Economics University of Pennsylvania Philadelphia Pennsylvania USA; ^16^ University College London London UK

**Keywords:** Enhanced Recovery After Surgery, ERAS, major trauma, perioperative care, polytrauma

## Abstract

**Background:**

Enhanced recovery after surgery (ERAS) protocols reduce length of stay, complications, and costs for elective surgical procedures. It remains challenging to implement ERAS concepts in the acute trauma patient due to deranged physiological reserve from the penetrating or blunt trauma producing altered physiology. However, systems of care improve access to early intervention and potentially reduce mortality. These consensus guidelines examine optimal pre‐hospital, resuscitation‐room, intra‐ and post‐operative treatment, systems of ethical management, and overall care for trauma patients in the post‐resuscitation phase of care. The guideline is presented in three parts, this being part 3.

**Methods:**

Experts in aspects of management of trauma surgical patients and intensive care were invited to contribute by the International ERAS Society and IATSIC. Pubmed, Cochrane, Embase, and MEDLINE database searches on English language publications were performed for ERAS elements using the patient intervention comparator outcome (PICO) consensus questions created by the expert group. Studies were selected with particular attention to randomized clinical trials, systematic reviews, meta‐analyses, and large cohort studies; reviewed; and summarized recommendations were graded using the grading of recommendations, assessment, development and evaluation (GRADE) system. These recommendations based on current best evidence, with extrapolation from elective patient studies where appropriate, were followed by a modified two‐round Delphi method to validate final recommendations. Several ERAS components are already standard of care within national and society guidelines and are endorsed. The bulk of the text focuses on key areas pertaining specifically to trauma care of major trauma and polytrauma in the ICU‐requiring group.

**Results:**

Overall 37 aspects of trauma care were considered with multiple PICO questions and sub‐points. Consensus was reached after two rounds of a modified Delphi process involving all authors, with minor adjustments to some phrasing required but with 87% overall agreement on all statements (100% agreement on 31 of the main statement sets, prior to minor edits to address the points of difference for the rest with 100% total agreement thereafter). None were rejected outright. The recommendations and level of evidence for each aspect of trauma care that may impact on improved recovery and reduced length of hospital stay are presented with grade of recommendation.

**Conclusions:**

Four main areas of relevance to ethics and systems are presented as part 3. The guidelines are based on current best evidence for an ERAS approach to patients who have had major injuries and polytrauma. These guidelines are not exhaustive but collate the best available evidence on important components of care for this patient population. As some of the evidence is extrapolated from elective surgery and non‐trauma emergency surgery, some of the components need further evaluation in future studies.

## 1 Introduction

In this third part of the ERAS/IATSIC guidelines the aspects relating to ethical dilemmas in the trauma population and various systems issues are addressed. These dovetail with general trauma systems publications; however, these guidelines address all income spectrums and not just high‐income well‐resourced environments. The background was presented and the methods were detailed in paper 1. We refer the reader to Parts 1 and 2 of this set of guidelines for the more clinical care aspects. (The papers are found as https://doi.org/10.1002/wjs.70002 and https://doi.org/10.1002/wjs.70004 in the *World Journal of Surgery*.)

33. Ethics in ICU

33a. Palliative care

**PICO: For trauma patients what is the best practice to provide advanced palliative care?**

Devastating injuries require both urgent assessment by a trauma service and early attention to patients' goals of care (GOC). The American College of Surgeons Trauma Quality Improvement Program (ACS‐TQIP) evidence‐based guidelines recommend an initial palliative assessment within 24 h of admission and family meeting, if needed, within 72 h to determine patient wishes and GOC [1]. Despite this, there is considerable worldwide variability in decisions to withhold/withdraw life‐sustaining treatments [2]. Although most intensivists reported that palliative care was beneficial, most note that it is underutilized [3].

Essential components of post‐trauma palliative care include effective communication and support around prognosis, and treatment options; psychosocial, emotional, and spiritual care; early and continuous assessment and treatment of pain, discomfort, and anxiety; and ongoing support of the patient and family. Although all trauma patients in the ICU should undergo screening for palliative care needs, certain considerations increase the likelihood that a palliative care intervention is indicated including severe traumatic injury with either the high risk of in‐hospital mortality, permanent disability, or functional outcome incompatible with patient's wishes. Prior health and functional status should be assessed [4].

Of the many hypothesized predictors of palliation, injury severity was the only tangible independent predictor. A persistent disconnect exists between the patient's wishes via living wills or advanced directives ‘in a terminal condition’ and fulfillment during end‐of‐life (EOL) decision‐making. This speaks to the complex nature of EOL decisions and the need for a multidisciplinary approach [5]. Many injured patients or their families make the difficult decision to withdraw life‐sustaining therapies (WLST) following severe injury [5]. WLST is common after severe TBI, a decision that may be compounded by socioeconomic factors with variation based on race, payment, and region [6, 7].

Elderly injured patients with significant comorbidities may also benefit from early palliative care. One study demonstrated that palliative care utilization was very high for older trauma patients who died in hospital. In contrast, the majority of those who were discharged alive, but with poor outcomes, did not have palliative care [8]. Interestingly, almost one‐third of patients with a decision to withhold/withdraw life‐sustaining treatment left the hospital alive [2]. Although most trauma surgeons reported palliative care beneficial, those surveyed indicate that palliative care is underutilized. Barriers identified provide important opportunities to further appropriate utilization of palliative care services [3]. Predictors of effective and consistent PC implementation include trauma center designation status and closed intensive care units [8]. Implementation of the American College of Surgeons TQIP palliative guidelines allows for an effective primary palliative care approach, selectively consulting specialty palliative care only when needed [9]. Defined order sets may also increase compliance with palliative care implementation [1, 10]. A multidisciplinary approach including intensivists, surgeons, and palliative care improves effective utilization of palliative care [11].

*33a. Summary and recommendations:*

Early implementation of palliative care in patients with severe injury with an anticipated high risk of hospital mortality, permanent disability, or functional outcome incompatible with patient's wishes is advised. Prior health and pre‐injury functional status should be assessed. Standardized screening protocols and order sets increase compliance with timely palliative care intervention. We recommend an initial palliative assessment within 24 h of admission and a family meeting, if needed, within 72 h of admission to the ICU.

Level of evidence: Moderate

Strength of recommendation: Strong

33b. Do Not Resuscitate (DNR)

**33b. What is the best practice to review and implement DNR orders in the trauma patient?**

The purpose of a do not attempt resuscitation (DNAR) order is to prevent unnecessary and potentially harmful attempts of providing a therapy that may not be beneficial, especially in patients with an irreversible or terminal clinical condition [12, 13, 14]. The incidence varies from 5% to 15% according to the literature [15]. However, at times, patients with DNAR orders become candidates for surgical procedures that may provide significant a therapeutic benefit, even though the procedure may not change the natural history of their underlying disease. This may especially be the case in trauma patients where the clinical presentation may be acute, unexpected, and perhaps not specific to the intent of the original DNAR order.

Historically, full revocation of perioperative DNAR was accepted, especially among polytrauma patients in an ‘emergency’ situation. Examples included auto enforcement, misinterpretation of intentions, and a one‐size‐fits‐all approach that may not have always aligned with patient preferences. Such approaches undermine the goal of shared decision‐making [16, 17].

Even in time sensitive circumstances, it is best practice to provide the patient or their designated surrogate, a clear discussion of the proposed treatment, therapeutic and non‐therapeutic options in the event of an intraoperative cardiac arrest, and the opportunity to refuse treatment in the event of an arrest. It is imperative to also document the conversation in the patient's medical record. The conversation between the patient, anesthesia, and surgeon should be clear without ambiguity. In the event of an agreeable temporary suspension of DNAR, it should be well documented when the perioperative period ends (e.g., immediately post‐operative or within 24 h [18]).

Surgical, anesthesia, and nursing societies have all published consensus guidelines around DNAR [19, 20, 21, 22, 23]. Ethically, they each highlight shared decision‐making, the importance of clear communication to assure patient wishes are respected, and the concept of required validation of existing DNAR orders to determine if they still apply to the clinical situation. Such required reconsideration is acceptable and requires the active engagement of the surgeon in the care of the patient. Specific DNAR options include full suspension of existing DNAR during the procedure and immediate postoperative period, limiting interventions (e.g., chest compressions), and deferment of decisions to the clinical team judgment based on the context and how it may or may not align with the preoperative patient goals. Regardless, the surgeon must remain actively engaged with clear language to best support the patient.

*33b. Summary and recommendations:*

We recommend implementation of DNAR orders in the trauma patient when appropriate and within the confines of local legal policies. This includes required validation or reconsideration, clear communication, and a timeline for DNAR reinitiation if it is temporarily suspended.

Level of evidence: High

Level of recommendation: Strong

33c. Advanced Care Planning

**PICO: In trauma patients with severe injury, what is the recommended approach to advanced care planning, whether for palliation or otherwise?**

Advanced care planning (ACP) is the process of discussing and documenting patients' preferences and values for future medical care, especially in the event of life‐threatening illness or injury and for informing the patient or the family of the predicted course of progress and prognosis. ACP can improve the quality of care and reduce unwanted interventions, conflicts, and stress for patients, families, and health care providers. However, ACP is often neglected or delayed for trauma patients, who may face sudden and unpredictable situations that require complex and time‐sensitive decisions [24, 25, 26, 27].

Barriers to ACP can be patient‐related, provider‐related, and system‐related factors. The most common patient‐related barriers include lack of awareness or understanding of ACP, reluctance or difficulty to discuss end‐of‐life issues, uncertainty or variability of preferences, and cultural or religious beliefs. The most common provider‐related barriers include lack of time or opportunity, lack of training or confidence, lack of communication or collaboration, and ethical or legal concerns. The most common system‐related factors include ACP champions, provision of education, use of standardized protocols or guidelines, cultural variability, and recognition of triggers or cues. All patients should be screened and those with poor prognosis or other factors that may require care limitation proceed to a formal process.

Identification of advanced care plans should begin at the entry portal of the emergency resuscitation room. Triggers to identify patient care preferences should be utilized, especially in healthcare systems that support pre‐arrival patient care preference. One retrospective study demonstrated the opportunities to avoid unwanted care in concordance with patient preference and develop a protocolized approach [24]. Advanced care planning before major surgery appears important to surgeons and families but continues to be rarely discussed [25, 26]. For those at high risk of mortality, palliative care services are becoming more available. Despite this additional resource, it is often an underutilized service to support families and facilitate advanced care planning. One recent retrospective study of trauma patients demonstrated that only 6.2% of eligible elderly patients received a palliative care consultation and 5% had any ACP documentation identified [26].

Based on the trauma quality improvement project (TQIP), Hwang et al. found that implementation of the TQIP best practices for palliative care and advanced care planning resulted in improvement in documentation of a GOC discussion within 72 h (17%–83%, *p* < 0.0001) and DNR orders placed more frequently (9% vs. 17%, *p* = 0.098) [27].

*33c. Summary and recommendation:*

Implementation of advanced care planning on admission and within 72 h of hospitalization is recommended. This can facilitate concordance of patient care wishes, family engagement and appropriate patient focused care. Implementation results in a higher proportion of pre‐existing decisions being documented and reflected in future care of the trauma patient and avoidance of unwanted care.

Level of evidence: Moderate

Strength of recommendation: Strong

34. Frailty index

**34. PICO In frail and elderly trauma patients, what is the best assessment and treatment approach to optimize outcome?**

Frail and elderly trauma patients are a vulnerable population that require special attention and care. They have higher mortality and morbidity rates, longer hospital stays, and lower functional outcomes than younger trauma patients. Frail and elderly trauma patients have complex and diverse needs that require a holistic and comprehensive approach to assessment and treatment with the involvement of geriatricians and hospital specialists [28, 29, 30, 31]. The best practice for this population is based on the following principles: early identification, multidisciplinary collaboration, patient‐centered care, and continuous evaluation and improvement.

Multiple objective measure scales may be utilized to screen frail and elderly trauma patients [28, 29]. Early identification of frail and elderly trauma patients is crucial for providing appropriate and timely interventions and preventing adverse outcomes. Examples of validated screening tools include the trauma‐specific frailty index (TSFI) or comprehensive geriatric assessment (GCA) that may help to identify patients who are at high risk of frailty and poor prognosis. The TSFI frailty index (odds ratio = 1.5; 95% CI: 1.1–2.5) is a significant predictor for unfavorable discharge disposition [30].

Multidisciplinary collaboration is essential for delivering optimal and coordinated care for frail and elderly trauma patients. The involvement of a geriatric trauma service (GTS) can facilitate the communication and integration of various disciplines such as surgery, emergency medicine, geriatrics, nursing, pharmacy, physiotherapy, occupational therapy, social work, and nutrition. The GTS can also provide education and training for staff and implement evidence‐based protocols and guidelines for the management of this population. Trauma patients that receive geriatric trauma consultation are more likely to be discharged home (OR: 2.01 [95% CI: 1.24–3.24]) [31].

*34. Summary and recommendations:*

Trauma specific frailty screening scores are useful in advanced care planning. We cannot recommend one specific scoring system over another, *but do recommend that an objective measure should be utilized*. When possible, a geriatric trauma consultative service should be utilized to improve the best possible outcomes for elderly and frail patients.

Level of evidence: Moderate

Grade of recommendation: Strong

35. Post‐Traumatic Stress Disorder after major trauma

**35. PICO: In patients who survive major trauma or polytrauma what is the risk of PTSD and how can this be prevented and treated?**

Post‐traumatic stress disorder (PTSD) is not uncommon after major physical trauma (severe trauma and polytrauma) and is not a new phenomenon. First elucidated in 2008 in Australia it appears to be more associated with causal blame assignment than the actual injury severity [32]. As much as 20%–60% of all victims of major trauma will experience some degree of PTSD at 12 months post injury [33, 34, 35, 36]. Risk factors, including female sex, age, low Glasgow scores, and penetrating trauma more than blunt trauma, along with ICU admission and longer hospitalization, predicted PTSD and depression [37]. Screening tools such as the post‐traumatic adjustment screen (PAS) were shown to be useful in identification of these patients within 6 months of hospital discharge [38]. Prevention is challenging and treatment appears to be less effective with pharmacological intervention compared to various forms of psychological intervention and support, although anti‐psychotics and prazosin appear to be opportune medical therapies to consider based on systematic review evidence [39, 40]. Ketamine, orally or intravenously, is emerging as a favored additional medical therapy for PTSD [41, 42]. Benzodiazepines worsen the risk of PTSD and alpha‐blockers and certain SSRI (paroxetine) and SNRI drugs including sertraline or quetiapine may be useful [43, 44, 45]. There does not appear to be robust evidence of any effective prevention therapy post‐polytrauma to reduce the risk of developing PTSD.

*35. Summary and recommendations:*

PTSD is common at 6–12 months post major trauma and treatment is a combination of psychotherapy, and pharmacotherapy adjusted to response. Prevention is difficult, but screening is recommended.

Level of evidence: Moderate

Level of recommendation: Strong

36. Systems and organizational aspects

**36a. PICO: What is the evidence of benefit of trauma systems and does centralization of trauma offer advantages in outcomes?**

Trauma Systems were first promulgated in the late 1980's by Trunkey and have been established in varying degrees and with different philosophical approaches across many nations [46]. The concept of a trauma system includes everything from coordinated pre‐hospital care, emergency department care by trauma‐specific teams with clear trauma team activation criteria, immediate access to operating room facilities and the ICU, plus the associated allied health care and rehabilitation services to optimally provide return to active lifestyle [47, 48]. Whether there is a real‐world benefit to established trauma systems remains a point of contention, given that the majority of studies look at a single system or country and the general outcome of these studies is that there is low to moderate quality evidence of benefit to improved survival for the most severely injured patients [49, 50, 51, 52, 53, 54, 55].

Centralization of services appears to have both benefits to patient outcome for the most severely injured, yet may adversely affect the confidence (and skills) of practitioners not working in designated trauma centers in terms of the decision‐making and treatment of moderate and less severe trauma [53, 56]. Therefore, it appears from the low‐quality, but more recent, literature that inclusive trauma systems (including the entire healthcare spectrum) are better than exclusive systems (all trauma to a central facility) as the workload is spread efficiently and the most severely injured reach the highest level of care in an appropriate timeframe [57, 58, 59, 60, 61, 62]. Inclusive systems spread the workload based on severity across a number of facilities and allow the most severely injured to be treated at the correct level of care, whereas the more traditional exclusive systems direct all patients to a central treatment facility irrespective of severity. The latter are challenging to implement in LMICs with distance to centralized facilities and lack of human resources.

**36b. PICO: What is the status of development of ideal trauma systems versus real life in different countries, with different health systems, including in LMICs.**

For most LMIC's the development of trauma systems are in their infancy, but must be designed around the local health care system capability and not simply by copying HIC trauma care models [63, 64, 65, 66, 67]. The guidelines developed by WHO and various national trauma professional bodies, aimed at all levels of economic development, should guide the development of systems appropriate to the region or country [68, 69, 70, 71, 72, 73]. The generic elements are such that improvement can be made in any resource setting.

**36c. PICO: What is the optimal time to transfer for rural and other trauma patients in non‐trauma hospitals to avoid under‐ or overtriage? Does aeromedical transport offer benefits?**

Whereas trauma is best managed by experts, rural patients and those who are admitted to ‘non‐designated’ trauma hospitals appear to have delayed access to care and worse related outcome measures [74, 75, 76, 77, 78, 79]. This requires early identification of the major trauma or polytrauma patient, appropriate optimizing of physiology and rapid transfer to definitive care; however, the latter may take some time to achieve and may avoid transfer completely in mature inclusive systems [80]. Delaying transfer through awaiting imaging or non‐essential surgical intervention is not advocated.

Triage using pre‐defined criteria aim to get the most severely injured patient to the most advanced care facility in the shortest timeframe and this has been shown to improve outcome; however, this often leads to overtriage and may necessitate unnecessary interfacility transfers leading to system overload at the pinnacle of the system—the level 1 (or equivalent) trauma center [81]. Secondary overtriage is a problem both with scene calls (often because of distance) and rurality for interfacility transfers [82, 83, 84, 85, 86]. By using system criteria this overtriage can be reduced, while also ensuring undertriage does not occur, this is true for both HIC and LMIC, and tools exist to assess the extent of the mistriage [87, 88, 89, 90]. This triage issue applies especially to older adults and children [91, 92, 93, 94].

Current high‐quality methodology systematic reviews and an in‐process EPOC‐Cochrane review suggest there remains low‐quality evidence for the effectiveness of inclusive trauma systems (over exclusive systems) and very‐low‐quality evidence for aeromedical injury mortality reduction, but timely transfer in rural and other extreme locations, compared to limited benefit in urban environments. Research should be directed to the recommended components of trauma systems and non‐fatal outcomes and explore whether system component interactions improve outcomes [44, 95, 96, 97, 98, 99, 100]. There remains much potential for improvement of the systems and it is important that the trauma system is relevant to the environment in which it operates, be that HIC or LMIC scenarios [101].

**36d. PICO: Does police and public scoop and run improve outcomes as compared to waiting for Emergency Services?**

Where urban penetrating trauma is prevalent, there is moderate evidence that for certain mechanisms (penetrating chest trauma and gunshot wounds), waiting for EMS is not beneficial and police or other personal vehicle transport to the nearest trauma center is associated with better survival [102, 103, 104].

**36e. What systems of care should be offered for the older trauma patient with major trauma or polytrauma?**

Trauma in the older patient is associated with many challenges, not least of which is the risk of under‐triage by health care providers from the scene to the hospital level of care. The existing guidelines do identify the most at‐risk patients, although triage tools may miss severe trauma in the older patient [105, 106, 107, 108]. This appears to be a national experience across the USA [109, 110]. The older patient may not show the usual physiological parameters that clinically indicate major trauma [111]. This may be due to polypharmacy and age‐related anatomical and physiological complexities [112]. Once identified, however it appears from the evidence that the appropriate level of care is best provided in a trauma system that is comprehensive and inclusive [113, 114]. Using appropriate physiological parameters in the older patient, for example systolic pressure of 110mmHg, improves the identification of those at risk [114].

Frailty scores and injury patterns should be aligned with age and physiology to dictate both treatment and where indicated palliation [28]. Transferring those who require a higher level of care is enhanced by the use of scoring systems [115].

*36. Summary and recommendations:*

Trauma systems do improve outcome but require the entire inclusive system to cooperate for the benefit of the patient. Systems must be developed that are locally relevant and that do not simply copy HIC models as the only option. However, the generic elements are the same. Triage to the correct level of care avoids overloading the system, whereas for select injuries direct transfer avoiding EMS may improve outcome. Rural aeromedical systems are beneficial but not urban helicopter transport.

36a. and 36b. Level of evidence: Trauma systems and development/maturity: Moderate

Grade of recommendation: Strong

36c. Level of evidence: Triage, transfer to centralized care and aeromedical transfer: Moderate

Grade of recommendation: Strong

36d. Level of evidence: Non‐EMS transport penetrating trauma: Low

Grade of recommendation: Weak

36e. Level of evidence: Moderate

Grade of recommendation: Strong

37. Pediatric‐specific considerations

37. PICO: Pediatric considerations are inserted where appropriate in main text. **What is the role of pediatric trauma centers vs pediatric trauma care in adult trauma centers? What is the ideal versus real life across the world?**

Appropriate treatment of pediatric trauma requires a multidisciplinary approach based on the anatomic, physiologic and social needs of children. On that background, the American College of Surgeons‐Committee on Trauma championed the creation of specific pediatric trauma centers [116]. Whether children treated in pediatric trauma centers have better outcomes than those treated in adult or combined (pediatric and adult) trauma centers is still a matter of discussion, provided guidelines are followed [117]. Such a debate has limited global relevance since the vast majority of pediatric trauma, the United States included, are still treated in hospitals focused on treating adults [118]. As previously mentioned, in most LMICs trauma system development is in an early phase and must be built on the local health care resources and not simply by copying HIC trauma care models.

*37. Summary and recommendations:*

There is no good evidence to support pediatric‐specific trauma centers outside a few high‐income countries and the local system must determine the most appropriate care for children.

37. Level of evidence: Moderate

Grade of Evidence: Weak

## 2 Conclusion

This third part of the ERAS/IATSIC enhanced recovery after major or polytrauma guidelines has presented the aspects related to ethical decision‐making advanced care planning, and long‐term complications as well as addressed certain aspects of trauma systems across the world. This concludes the three parts of this ERAS/IATSIC guideline.

## Author Contributions

**Timothy C. Hardcastle:** conceptualization, methodology, data curation, investigation, formal analysis, writing – original draft, writing – review and editing. **Christine Gaarder:** writing – original draft, writing – review and editing, conceptualization, methodology, data curation, formal analysis, investigation. **Zsolt Balogh:** data curation, investigation, formal analysis, writing – review and editing. **Scott D'amours:** data curation, investigation, formal analysis, writing – review and editing. **Kimberly A. Davis:** data curation, investigation, formal analysis, writing – review and editing. **Amit Gupta:** data curation, investigation, formal analysis, writing – review and editing. **Shahin Mohseni:** methodology, data curation, investigation, formal analysis, writing – review and editing. **Paal A. Naess:** investigation, data curation, writing – review and editing, formal analysis. **Shanisa Naidoo:** investigation, formal analysis, data curation, writing – review and editing. **Tarek Razek:** data curation, investigation, formal analysis, writing – review and editing. **Simon Robertson:** data curation, investigation, formal analysis, writing – review and editing. **Hayaki Uchino:** investigation, data curation, formal analysis, writing – review and editing. **David Zonies:** data curation, investigation, formal analysis, writing – review and editing. **Jade Whing:** project administration, data curation, funding acquisition, writing – review and editing, formal analysis. **Michael J. Scott:** methodology, data curation, investigation, formal analysis, supervision, writing – review and editing, writing – original draft.

## Conflicts of Interest

Timothy C. Hardcastle, Christine Gaarder, Zsolt Balogh, Scott D'amours, Kimberly A. Davis, Amit Gupta, Shahin Mohseni, Paal A. Naess, Shanisa Naidoo, Tarek Razek, Simon Robertson, Hayaki Uchino, David Zonies, and Jade Whing have no conflicts of interest. Dr Michael J. Scott, representing the ERAS group, has honoraria from and serves on advisory boards of Baxter, Edwards Lifesciences, Deltex, Trevena, and Merck. He also receives travel reimbursement from these companies and is Past President of ERAS USA.

## Data Availability

Data sharing is not applicable to this article as no new data were created or analyzed in this study.

## References

[1] ACS TQIP Palliative Care Best Practices Guidelines, accessed September 16, 2024, https://www.facs.org/media/g3rfegcn/palliative_guidelines.pdf.

[2] S. M. Lobo , F. H. B. De Simoni , S. M. Jakob , et al.,“Decision‐Making on Withholding or Withdrawing Life Support in the ICU: A Worldwide Perspective,” Chest 152, no. 2 (2017): 321–329, 10.1016/j.chest.2017.04.176. 28483610

[3] M. Karlekar , B. Collier , A. Parish , L. Olson , and T. Elasy , “Utilization and Determinants of Palliative Care in the Trauma Intensive Care Unit: Results of a National Survey,” Palliative Medicine 28, no. 8 (2014): 1062–1068, 10.1177/0269216314534514. 24827834

[4] M. Wooster , A. Stassi , J. Hill , J. Kurtz , M. Bonta , and M. C. Spalding , “End‐of‐Life Decision‐Making for Patients With Geriatric Trauma Cared for in a Trauma Intensive Care Unit,” American Journal of Hospice and Palliative Medicine 35, no. 8 (2018): 1063–1068, 10.1177/1049909117752670. 29366336

[5] J. M. Leonard , S. F. Polites , N. D. Martin , A. E. Glasgow , E. B. Habermann , and L. J. Kaplan , “Comfort Care in Trauma Patients Without Severe Head Injury: In‐Hospital Complications as a Trigger for Goals of Care Discussions,” Injury 50, no. 5 (2019): 1064–1067, 10.1016/j.injury.2019.01.024. 30745124

[6] T. Williamson , M. D. Ryser , P. A. Ubel , et al., “Withdrawal of Life‐Supporting Treatment in Severe Traumatic Brain Injury,” JAMA Surgery 155, no. 8 (2020): 723–731, PMID: 32584926, 10.1001/jamasurg.2020.1790. 32584926 PMC7301301

[7] G. Hoit , D. N. Wijeysundera , D. M. Hamad , et al., “Insurance Type and Withdrawal of Life‐Sustaining Therapy in Critically Injured Trauma Patients,” JAMA Network Open 7, no. 7 (2024): e2421711, 10.1001/jamanetworkopen.2024.21711. 39046743 PMC11270131

[8] M. Fiorentino , F. Hwang , S. R. Pentakota , D. H. Livingston , and A. C. Mosenthal , “Palliative Care in Trauma: Not Just for the Dying,” Journal of Trauma and Acute Care Surgery 87, no. 5 (2019): 1156–1163, 10.1097/TA.0000000000002440. 31658239

[9] A. Edsall , S. Howard , E. N. Dewey , et al., “Critical Decisions in the Trauma Intensive Care Unit: Are We Practicing Primary Palliative Care?,” Journal of Trauma and Acute Care Surgery 91, no. 5 (2021): 886–890, 10.1097/TA.0000000000003324. 34695065

[10] P. D. Treece , R. A. Engelberg , L. Crowley , et al., “Evaluation of a Standardized Order Form for the Withdrawal of Life Support in the Intensive Care Unit,” Critical Care Medicine 32, no. 5 (2004): 1141–1148, 10.1097/01.ccm.0000125509.34805.0c. 15190964

[11] J. Wycech , A. A. Fokin , J. K. Katz , et al., “Reduction in Potentially Inappropriate Interventions in Trauma Patients Following a Palliative Care Consultation,” Journal of Palliative Medicine 24, no. 5 (2021): 705–711, 10.1089/jpm.2020.0218. 32975481

[12] L. R. Hopson , E. Hirsh , J. Delgado , R. M. Domeier , N. E. McSwain , and J. Krohmer , “Guidelines for Withholding or Termination of Resuscitation in Prehospital Traumatic Cardiopulmonary Arrest: Joint Position Statement of the National Association of EMS Physicians and the American College of Surgeons Committee on Trauma,” Journal of the American College of Surgeons 196, no. 1 (2003): 106–112, 10.1016/s1072-7515(02)01668-x. 12517561

[13] W. Teeter and D. Haase , “Updates in Traumatic Cardiac Arrest,” Emergency Medicine Clinics of North America 38, no. 4 (2020): 891–901, 10.1016/j.emc.2020.06.009. 32981624

[14] American College of Surgeons Committee on Trauma, American College of Emergency Physicians Pediatric Emergency Medicine Committee, National Association of EMS Physicians , et al., “Withholding or Termination of Resuscitation in Pediatric Out‐of‐Hospital Traumatic Cardiopulmonary Arrest,” Annals of Emergency Medicine 63, no. 4 (2014): 504–515, 10.1016/j.annemergmed.2014.01.013. 24655460

[15] K. Salottolo , P. J. Offner , A. Orlando , et al., “The Epidemiology of Do‐Not‐Resuscitate Orders in Patients With Trauma: A Community Level One Trauma Center Observational Experience,” Scandinavian Journal of Trauma, Resuscitation and Emergency Medicine 23, no. 1 (2015): 9, 10.1186/s13049-015-0094-2. 25645242 PMC4333154

[16] C. M. Burkle , P. S. Mueller , K. M. Swetz , C. C. Hook , and M. T. Keegan , “Physician Perspectives and Compliance With Patient Advance Directives: The Role External Factors Play on Physician Decision Making,” BMC Medical Ethics 13, no. 1 (2012): 31, 10.1186/1472-6939-13-31. 23171364 PMC3528447

[17] N. M. Mollberg , S. R. Wise , K. Berman , et al., “The Consequences of Noncompliance With Guidelines for Withholding or Terminating Resuscitation in Traumatic Cardiac Arrest Patients,” Journal of Trauma 71, no. 4 (2011): 997–1002, 10.1097/TA.0b013e3182318269. 21986740

[18] N. S. Wenger , N. L. Greengold , R. K. Oye , et al., “Patients With DNR Orders in the Operating Room: Surgery, Resuscitation, and Outcomes. SUPPORT Investigators. Study to Understand Prognoses and Preferences for Outcomes and Risks of Treatments,” Journal of Clinical Ethics 8, no. 3 (1997): 250–257, PMID: 9436083, 10.1086/jce199708305. 9436083

[19] British Medical Association, Resuscitation Council UK, Royal College of Nursing , Decisions Relating to Cardiopulmonary Resuscitation, 3rd Revision (2016), https://www.resus.org.uk/library/publications/publication‐decisions‐relating‐cardiopulmonary. 10.1136/jme.27.5.310 PMC1733441 11579186

[20] W. W. Kridelbaugh , “‘Do Not Resuscitate’ in the Operating Room,” Bulletin of the American College of Surgeons 79, no. 9 (1994): 29–33, PMID: 10136710. 10136710

[21] AORN , Position Statement: Perioperative Care of Patients With Do‐Not‐Resuscitate or Allow‐Natural‐Death Orders (1995), http://www.aorn.org/PracticeResources/AORNPositionStatements/.

[22] ASA , Ethical Guidelines for the Anesthsia Care of Patients With Do‐Not‐Resuscitate Orders or Other Directives That Limit Treatment (1991), https://www.asahq.org/standards‐and‐practice‐parameters/statement‐on‐ethical‐guidelines‐for‐the‐anesthesia‐care‐of‐patients‐with‐do‐not‐resuscitate‐orders.

[23] M. Bawazeer , H. Al Alawyat , and M. Zamakhshary , “Applicability of Adult Guidelines for Withholding or Terminating Resuscitation for Prehospital Traumatic Cardiopulmonary Arrest in Pediatrics,” European Journal of Pediatric Surgery 25, no. 2 (2015): 206–211, 10.1055/s-0033-1357750. 24399668

[24] J. H. Ballou , E. N. Dewey , and D. H. Zonies , “Elderly Patients Presenting to a Level I Trauma Center With Physician Orders for a Life‐Sustaining Treatment Form: A Propensity‐Matched Analysis,” Journal of Trauma and Acute Care Surgery 87, no. 1 (2019): 153–160, 10.1097/TA.0000000000002321. 31033897

[25] E. Kalbfell , A. Kata , A. S. Buffington , et al., “Frequency of Preoperative Advance Care Planning for Older Adults Undergoing High‐Risk Surgery: A Secondary Analysis of a Randomized Clinical Trial,” JAMA Surgery 156, no. 7 (2021): e211521, 10.1001/jamasurg.2021.1521. 33978693 PMC8117055

[26] L. Haines , W. Wang , M. Harhay , N. Martin , S. Halpern , and K. Courtright , “Opportunities to Improve Palliative Care Delivery in Trauma Critical Illness,” American Journal of Hospice and Palliative Medicine 39, no. 6 (2022): 633–640, 10.1177/10499091211042303. 34467775 PMC8885767

[27] F. Hwang , J. Son , K. Ensor , et al., “Initiating Advance Care Planning at Admission: A Brief Intervention to Increase Goals of Care Discussions in Geriatric Trauma Patients in an Urban Level I Trauma Center,” Trauma Surgery & Acute Care Open 8, no. 1 (2023): e001058, 10.1136/tsaco-2022-001058. 38020856 PMC10660418

[28] A. Thompson , S. Gida , Y. Nassif , C. Hope , and A. Brooks , “The Impact of Frailty on Trauma Outcomes Using the Clinical Frailty Scale,” European Journal of Trauma and Emergency Surgery 48, no. 2 (2022): 1271–1276, 10.1007/s00068-021-01627-x. 33682027 PMC7937544

[29] A. Poulton , J. F. Shaw , F. Nguyen , et al., “The Association of Frailty With Adverse Outcomes After Multisystem Trauma: A Systematic Review and Meta‐Analysis,” Anesthesia & Analgesia 130, no. 6 (2020): 1482–1492, 10.1213/ANE.0000000000004687. 32384338

[30] B. Joseph , V. Pandit , B. Zangbar , et al., “Validating Trauma‐Specific Frailty Index for Geriatric Trauma Patients: A Prospective Analysis,” Journal of the American College of Surgeons 219, no. 1 (2014): 10–17e1, 10.1016/j.jamcollsurg.2014.03.020. 24952434

[31] J. Lampron , L. Khoury , J. Moors , et al., “Impact of a Geriatric Consultation Service on Outcomes in Older Trauma Patients: A Before‐After Study,” European Journal of Trauma and Emergency Surgery 48, no. 4 (2022): 2859–2865, 10.1007/s00068-021-01724-x. 34146122

[32] I. A. Harris , J. M. Young , H. Rae , B. B. Jalaludin , and M. J. Solomon , “Predictors of Post‐Traumatic Stress Disorder Following Major Trauma,” ANZ Journal of Surgery 78, no. 7 (2008): 583–587, 10.1111/j.1445-2197.2008.04578.x. 18593415

[33] A. Baranyi , O. Leithgob , B. Kreiner , et al., “Relationship Between Posttraumatic Stress Disorder, Quality of Life, Social Support, and Affective and Dissociative Status in Severely Injured Accident Victims 12 Months After Trauma,” Psychosomatics 51, no. 3 (2010): 237–247, 10.1176/appi.psy.51.3.237. 20484722

[34] T. H. Rainer , J. H. H. Yeung , S. K. C. Cheung , et al., “Assessment of Quality of Life and Functional Outcome in Patients Sustaining Moderate and Major Trauma: A Multicentre, Prospective Cohort Study,” Injury 45, no. 5 (2014): 902–909, 10.1016/j.injury.2013.11.006. 24314871

[35] A. H. Haider , J. P. Herrera‐Escobar , S. S. Al Rafai , et al., “Factors Associated With Long‐Term Outcomes After Injury: Results of the Functional Outcomes and Recovery After Trauma Emergencies (FORTE) Multicenter Cohort Study,” Annals of Surgery 271, no. 6 (2020): 1165–1173, 10.1097/SLA.0000000000003101. 30550382

[36] E. E. Spijker , K. Jones , J. W. Duijff , A. Smith , and G. R. Christey , “Psychiatric Comorbidities in Adult Survivors of Major Trauma: Findings From the Midland Trauma Registry,” Journal of Primary Health Care 10, no. 4 (2018): 292–302, 10.1071/HC17091. 31039958

[37] R. Ahl , R. Lindgren , Y. Cao , L. Riddez , and S. Mohseni , “Risk Factors for Depression Following Traumatic Injury: An Epidemiological Study From a Scandinavian Trauma Center,” Injury 48, no. 5 (2017): 1082–1087, 10.1016/j.injury.2017.03.019. 28356197

[38] L. Johnson , C. Lodge , S. Vollans , and P. J. Harwood , “Predictors of Psychological Distress Following Major Trauma,” Injury 50, no. 9 (2019): 1577–1583, 10.1016/j.injury.2019.05.031. 31196596

[39] M. Haerizadeh , J. A. Sumner , J. L. Birk , et al., “Interventions for Posttraumatic Stress Disorder Symptoms Induced by Medical Events: A Systematic Review,” Journal of Psychosomatic Research 129 (2020): 109908, 10.1016/j.jpsychores.2019.109908. 31884302 PMC7580195

[40] P. A. Coventry , N. Meader , H. Melton , et al., “Psychological and Pharmacological Interventions for Posttraumatic Stress Disorder and Comorbid Mental Health Problems Following Complex Traumatic Events: Systematic Review and Component Network Meta‐Analysis,” PLoS Medicine 17, no. 8 (2020): e1003262, 10.1371/journal.pmed.1003262. 32813696 PMC7446790

[41] H. A. MacConnel , M. Earleywine , and S. Radowitz , “Rapid and Sustained Reduction of Treatment‐Resistant PTSD Symptoms After Intravenous Ketamine in a Real‐World, Psychedelic Paradigm,” Journal of Psychopharmacology 14, no. 1 (2024): 2698811241286726, 10.1177/02698811241286726. 39400075

[42] H. Kaur and C. Andrade , “Dramatic and Persistent Relief From Chronic Suicidality, Emotional Disturbances, and Trauma Symptomatology With Oral Ketamine and Memantine in Refractory Depression With Complex PTSD,” Indian Journal of Psychiatry 66, no. 9 (2024): 870–871, 10.4103/indianjpsychiatry.indianjpsychiatry_587_24. 39502586 PMC11534131

[43] T. Tregub , M. Lytvynenko , V. Kukushkin , et al., “Pharmacology of Post‐Traumatic Stress Disorder,” Georgian Medical News 342 (2023): 122–124, PMID: 37991966. 37991966

[44] Z.‐X. Zhang , R.‐B. Liu , J. Zhang , et al., “Clinical Outcomes of Recommended Active Pharmacotherapy Agents From NICE Guideline for Post‐Traumatic Stress Disorder: Network Meta‐Analysis,” Progress in Neuro‐Psychopharmacology & Biological Psychiatry 125 (2023): 110754, 10.1016/j.pnpbp.2023.110754. 36934999

[45] M. D. Hoskins , J. Bridges , R. Sinnerton , et al., “Pharmacological Therapy for Post‐Traumatic Stress Disorder: A Systematic Review and Meta‐Analysis of Monotherapy, Augmentation and Head‐to‐Head Approaches,” European Journal of Psychotraumatology 12, no. 1 (2021): 1802920, 10.1080/20008198.2020.1802920. 34992738 PMC8725683

[46] D. D. Trunkey , “Trauma Care Systems,” Emergency Medicine Clinics of North America 2, no. 4 (1984): 913–922, PMID: 6532788, 10.1016/s0733-8627(20)30901-9. 6532788

[47] R. A. Lendrum and D. J. Lockey , “Trauma System Development,” supplement, Anaesthesia 68, no. S1 (2013): 30–39, 10.1111/anae.12049. 23210554

[48] J. Zhou , T. Wang , I. Belenkiy , et al., “Management of Severe Trauma Worldwide. Implementation of Trauma Systems in Emerging Countries: China, Russia and South Africa,” Critical Care Medicine 25, no. 1–10 (2021): 286, 10.1186/s13054-021-03681-8. PMC8352140 34372903

[49] S. Dijkink , C. J. Nederpelt , P. Krijnen , G. C. Velmahos , and I. B. Schipper , “Trauma Systems Around the World: A Systematic Overview,” Journal of Trauma and Acute Care Surgery 83, no. 5 (2017): 917–925, 10.1097/TA.0000000000001633. 28715361

[50] J. S. David , P. Bouzat , and M. Raux , “Evolution and Organisation of Trauma Systems,” Aaesthesia, Critical Care & Pain Medicine 38, no. 2 (2019): 161–167, 10.1016/j.accpm.2018.01.006. 29476943

[51] K. W. W. Lansink and L. P. H. Leenen , “Do Designated Trauma Systems Improve Outcome?,” Current Opinion in Critical Care 13, no. 6 (2007): 686–690, 10.1097/MCC.0b013e3282f1e7a4. 17975391

[52] A. Ernstberger , M. Koller , F. Zeman , et al., “A Trauma Network With Centralized and Local Health Care Structures: Evaluating the Effectiveness of the First Certified Trauma Network of the German Society of Trauma Surgery,” PLoS One 13, no. 3 (2018): e0194292, 10.1371/journal.pone.0194292. 29538456 PMC5851627

[53] S. Magnone , A. Ghirardi , M. Ceresoli , and L. Ansaloni , “Trauma Patients Centralization for the Mechanism of Trauma: Old Questions Without Answers,” European Journal of Trauma and Emergency Surgery 45, no. 3 (2019): 431–436, 10.1007/s00068-017-0873-8. 29127439

[54] L. Moore , H. Champion , P.‐A. Tardif , et al., “Impact of Trauma System Structure on Injury Outcomes: A Systematic Review and Meta‐Analysis,” World Journal of Surgery 42, no. 5 (2018): 1327–1339, 10.1007/s00268-017-4292-0. 29071424

[55] M. Putland , M. Noonan , A. Olaussen , P. Cameron , and M. Fitzgerald , “Low Major Trauma Confidence Among Emergency Physicians Working Outside Major Trauma Services: Inevitable Result of a Centralised Trauma System or Evidence for Change?,” Emergency Medicine Australasia 30, no. 6 (2018): 834–842, 10.1111/1742-6723.13135. 30054972

[56] G. H. Tinkoff , J. F. Reed , R. Megargel , E. L. Alexander , S. Murphy , and M. S. Jones , “Delaware's Inclusive Trauma System: Impact on Mortality,” Journal of Trauma 69, no. 2 (2010): 245–252, 10.1097/TA.0b013e3181e493b9. 20699731

[57] S. Bokhari , N. Aslam‐Pervez , O. Riaz , Z. Sadozai , M. Bhamra , and P. Harwood , “What Effect Has the Major Trauma Network Had on Perceptions of Trauma Care Delivery Amongst Trauma Teams in Major Trauma Centres and Neighbouring Trauma Units?,” European Journal of Trauma and Emergency Surgery 47, no. 1 (2021): 171–177, 10.1007/s00068-019-01206-1. 31451862

[58] D. J. Galanis , S. Steinemann , L. Rosen , A. C. Bronstein , and W. L. Biffl , “Rural Level III Centers in an Inclusive Trauma System Reduce the Need for Interfacility Transfer,” Journal of Trauma and Acute Care Surgery 85, no. 4 (2018): 747–751, 10.1097/TA.0000000000002033. 30036262

[59] T. C. Hardcastle , M. Finlayson , M. van Heerden , B. Johnson , C. Samuel , and D. J. J. Muckart , “The Prehospital Burden of Disease Due to Trauma in KwaZulu‐Natal: The Need for Afrocentric Trauma Systems,” World Journal of Surgery 37, no. 7 (2013): 1513–1525, 10.1007/s00268-012-1852-1. 23196339

[60] T. C. Hardcastle , C. Samuels , and D. J. Muckart , “An Assessment of the Hospital Disease Burden and the Facilities for the In‐Hospital Care of Trauma in KwaZulu‐Natal, South Africa,” World Journal of Surgery 37, no. 7 (2013): 1550–1561, 10.1007/s00268-012-1889-1. 23250389

[61] C. R. Valcarcel , D. Bieler , G. A. Bass , et al., “ESTES Recommendations for the Treatment of Polytrauma—A European Consensus Based on the German S3 Guidelines for the Treatment of Patients With Severe/multiple Injuries,” European Journal of Trauma and Emergency Surgery 51, no. 1 (2025): 171, 10.1007/s00068-025-02852-4. 40214785 PMC11991986

[62] R. Pfeifer , F. Hildebrand , C. Gaarder , and I. Marzi , “European White Book on Polytrauma Management – Setting Standards for Trauma Care Across Europe,” European Journal of Trauma and Emergency Surgery 51, no. 1 (2025): 170, 10.1007/s00068-025-02834-6. 40205186 PMC11982066

[63] M. B. Mwandri and T. C. Hardcastle , “Evaluation of Resources Necessary for Provision of Trauma Care in Botswana: An Initiative for a Local System,” World Journal of Surgery 42, no. 6 (2018): 1629–1638, 10.1007/s00268-017-4381-0. 29185018

[64] M. Mwandri , T. C. Hardcastle , H. Sawe , et al., “Trauma Burden, Patient Demographics and Care‐Process in Major Hospitals in Tanzania: A Needs Assessment for Improving Healthcare Resource Management,” African Journal of Emergency Medicine 10, no. 3 (2020): 111–117, 10.1016/j.afjem.2020.01.010. 32923319 PMC7474232

[65] T. E. Callese , C. T. Richards , P. Shaw , et al., “Trauma System Development in Low‐ and Middle‐Income Countries: A Review,” Journal of Surgical Research 193, no. 1 (2015): 300–307, 10.1016/j.jss.2014.09.040. 25450600

[66] D. Bagaria , A. S. Ratnayake , A. Madrid , and T. J. Worlton , “Trauma Systems in Asian Countries: Challenges and Recommendations,” Critical Care 28, no. 1 (2024): 47, 10.1186/s13054-024-04838-x. 38365782 PMC10874018

[67] J. Whitaker , N. O'Donohoe , M. Denning , et al., “Assessing Trauma Care Systems in Low‐Income and Middle‐Income Countries: A Systematic Review and Evidence Synthesis Mapping the Three Delays Framework to Injury Health System Assessments,” BMJ Global Health 6, no. 5 (2021): e004324, 10.1136/bmjgh-2020-004324. PMC8118008 33975885

[68] C. Mock , J. Lormand , J. Goosen , M. Joshipura , and M. Peden , Guidelines for Essential Trauma Care (World Health Organization, 2004).

[69] Resources for the Optimal Care of Trauma, 2022, accessed August 8, 2024, https://www.facs.org/quality‐programs/trauma/quality/verification‐review‐and‐consultation‐program/standards/.

[70] Australia‐New Zealand Trauma Verification Program, accessed August 8, 2024, https://www.surgeons.org/research‐audit/trauma‐verification.

[71] I. Takeuchi , N. Morimura , M. Iwashita , et al., “Validating the Trauma Care System Developed by Yokohama City Local Government,” Acute Medicine & Surgery 9, no. 1 (2022): e749, 10.1002/ams2.749. 35462683 PMC9016721

[72] Major Trauma Care in England (National Audit Office), accessed August 8, 2024, https://www.nao.org.uk/report/major‐trauma‐care‐in‐england/.

[73] T. C. Hardcastle , E. Steyn , K. Boffard , et al., “Guideline for the Assessment of Trauma Centres for South Africa,” South African Medical Journal 101, no. 3 (2011): 189–194, 10.7196/samj.4682. 21382251

[74] N. R. Haslam , O. Bouamra , T. Lawrence , C. G. Moran , and D. J. Lockey , “Time to Definitive Care Within Major Trauma Networks in England,” BJS Open 4, no. 5 (2020): 963–969, 10.1002/bjs5.50316. 32644299 PMC7528529

[75] S. Tarima , A. Ertl , J. I. Groner , and L. D. Cassidy , “Factors Associated With Patients Transferred From Undesignated Trauma Centers to Trauma Centers,” Journal of Trauma and Acute Care Surgery 79, no. 3 (2015): 378–385, 10.1097/TA.0000000000000763. 26307869 PMC4552076

[76] K.‐T. Chen , H.‐C. Su , N.‐C. Wu , C.‐C. Hsu , and Yi Lin , “Clinical Features and Required Aids of Transferred Severe Trauma Patients,” J Acute Med 10, no. 3 (2020): 99–105, 10.6705/j.jacme.202009_10(3).0001. 33209568 PMC7662104

[77] K. Curtis , B. Kennedy , M. K. Lam , et al., “Pathways and Factors That Influence Time to Definitive Trauma Care for Injured Children in New South Wales, Australia,” Injury 53, no. 1 (2022): 61–68, 10.1016/j.injury.2021.02.036. 33632604

[78] R. M. Hasler , T. Rauer , H.‐C. Pape , and M. Zwahlen , “Inter‐Hospital Transfer of Polytrauma and Severe Traumatic Brain Injury Patients: Retrospective Nationwide Cohort Study Using Data From the Swiss Trauma Register,” PLoS One 16, no. 6 (2021): e0253504, 10.1371/journal.pone.0253504. 34143842 PMC8213144

[79] D. T. Harrington , M. Connolly , W. L. Biffl , S. D. Majercik , and W. G. Cioffi , “Transfer Times to Definitive Care Facilities Are Too Long: A Consequence of an Immature Trauma System,” Annals of Surgery 241, no. 6 (2005): 961–966, discussion 966‐968, 10.1097/01.sla.0000164178.62726.f1. 15912045 PMC1357175

[80] D. Gomez , A. S. Alali , W. Xiong , B. L. Zarzaur , N. C. Mann , and A. B. Nathens , “Definitive Care in Level 3 Trauma Centres After Severe Injury: A Comparison of Clinical Characteristics and Outcomes,” Injury 46, no. 9 (2015): 1790–1795, 10.1016/j.injury.2015.05.047. 26071325

[81] B. L. Gough , M. D. Painter , A. L. Hoffman , R. J. Caplan , C. A. Peters , and M. D. Cipolle , “Right Patient, Right Place, Right Time: Field Triage and Transfer to Level I Trauma Centers,” American Surgeon 86, no. 5 (2020): 400–406, 10.1177/0003134820918249. 32684018

[82] A.‐P. Deeb , H. M. Phelos , A. B. Peitzman , T. R. Billiar , J. L. Sperry , and J. B. Brown , “Geospatial Assessment of Helicopter Emergency Medical Service Overtriage,” Journal of Trauma and Acute Care Surgery 91, no. 1 (2021): 178–185, 10.1097/TA.0000000000003122. 33605701 PMC8243854

[83] R. G. Maine , C. Kajombo , G. Mulima , et al., “Secondary Overtriage of Trauma Patients to a Central Hospital in Malawi,” World Journal of Surgery 44, no. 6 (2020): 1727–1735, 10.1007/s00268-020-05426-0. 32100065

[84] B. M. Crowley , R. L. Griffin , W. Andrew Smedley , et al., “Secondary Overtriage of Trauma Patients: Analysis of Clinical and Geographic Patterns,” Journal of Surgical Research 254 (2020): 286–293, 10.1016/j.jss.2020.04.009. 32485430

[85] J. Con , D. Long , E. Sasala , et al., “Secondary Overtriage in a Statewide Rural Trauma System,” Journal of Surgical Research 198, no. 2 (2015): 462–467, 10.1016/j.jss.2015.03.077. 25959835 PMC4568987

[86] P. P. Parikh , P. Parikh , L. Mamer , M. C. McCarthy , and J. V. Sakran , “Association of System‐Level Factors With Secondary Overtriage in Trauma Patients,” JAMA Surgery 154, no. 1 (2019): 19–25, 10.1001/jamasurg.2018.3209. 30325989 PMC6439859

[87] K. Anderson , M. Schellenberg , N. Owattanapanich , et al., “Undertriage of Severely Injured Trauma Patients,” American Surgeon 89, no. 10 (2023): 4129–4134, 10.1177/00031348231177939. 37259503

[88] E. Jeppesen , M. Cuevas‐Østrem , C. Gram‐Knutsen , and O. Uleberg , “Undertriage in Trauma: An Ignored Quality Indicator?,” Scandinavian Journal of Trauma, Resuscitation and Emergency Medicine 28, no. 1 (2020): 3–4, 10.1186/s13049-020-00729-6. 32375842 PMC7204312

[89] B. Chin , N. Alter , D.‐D. Wright , et al., “Assessing Effectiveness and Efficiency of Need for Trauma Intervention (NFTI) and Modified NFTI in Identifying Overtriage and Undertriage Rates and Associated Outcomes,” American Surgeon 89, no. 12 (2023): 6181–6189, 10.1177/00031348231191225. 37480558

[90] M. A. Horst , S. Jammula , B. W. Gross , et al., “Undertriage in Trauma: Does an Organized Trauma Network Capture the Major Trauma Victim? A Statewide Analysis,” Journal of Trauma and Acute Care Surgery 84, no. 3 (2018): 497–504, 10.1097/TA.0000000000001781. 29283966

[91] D. C. Chang , R. R. Bass , E. E. Cornwell , and E. J. Mackenzie , “Undertriage of Elderly Trauma Patients to State‐Designated Trauma Centers,” Archives of Surgery 143, no. 8 (2008): 776–781, discussion 782, 10.1001/archsurg.143.8.776. 18711038

[92] A. C. Hoyle , L. C. Biant , and M. Young , “Undertriage of the Elderly Major Trauma Patient Continues in Major Trauma Centre Care: A Retrospective Cohort Review,” Emergency Medicine Journal 37, no. 8 (2020): 508–514, 10.1136/emermed-2019-208541. 32546474

[93] J. Holst , S. Perman , R. Capp , J. Haukoos , and A. Ginde , “Undertriage of Trauma‐Related Deaths in U.S. Emergency Departments,” Western Journal of Emergency Medicine 17, no. 3 (2016): 315–323, 10.5811/westjem.2016.2.29327. 27330664 PMC4899063

[94] J. Peng , K. Wheeler , J. I. Groner , K. J. Haley , and H. Xiang , “Undertriage of Pediatric Major Trauma Patients in the United States,” Clinical Pediatrics 56, no. 9 (2017): 845–853, 10.1177/0009922817709553. 28516800 PMC6518411

[95] J. H. Scaife , J. R. Bryce , S. E. Iantorno , M. Yang , M. L. McCrum , and B. T. Bucher , “Secondary Undertriage of Pediatric Trauma Patients Across the United States Emergency Departments,” Journal of Surgical Research 293 (2024): 37–45, 10.1016/j.jss.2023.07.054. 37703702

[96] L. Cobianchi , F. Dal Mas , M. Massaro , et al., “Team Dynamics in Emergency Surgery Teams: Results From a First International Survey,” World Journal of Emergency Surgery 16, no. 1 (2021): 47, 10.1186/s13017-021-00389-6. 34530891 PMC8443910

[97] L. Cobianchi , F. Dal Mas , M. Massaro , et al., “Diversity and Ethics in Trauma and Acute Care Surgery Teams: Results From an International Survey,” World Journal of Emergency Surgery 17, no. 1 (2022): 44, 10.1186/s13017-022-00446-8. 35948947 PMC9364511

[98] H. Wild , C. Marfo , C. Mock , et al., “Operative Trauma Courses: A Scoping Review to Inform the Development of a Trauma Surgery Course for Low‐Resource Settings,” World Journal of Surgery 47, no. 7 (2023): 1662–1683, 10.1007/s00268-023-06985-8. 36988651

[99] M. Mwandri , B. Stewart , T. C. Hardcastle , and R. L. Gruen , “Organised Trauma Systems and Designated Trauma Centres for Improving Outcomes in Injured Patients,” Cochrane Database of Systematic Reviews Issue 1 (2017), 10.1002/14651858.CD012500. PMC12315082 40747779

[100] S. M. Galvagno Jr. , R. Sikorski , J. M. Hirshon , et al., “Helicopter Emergency Medical Services for Adults With Major Trauma,” Cochrane Database of Systematic Reviews 2015, no. 12 (2015): CD009228, 10.1002/14651858.CD009228.pub3. 26671262 PMC8627175

[101] M. C. Reade , “Perspective: The Top 11 Priorities to Improve Trauma Outcomes, From System to Patient Level,” Critical Care 26, no. 1 (2022): 395, 10.1186/s13054-022-04243-2. 36544203 PMC9768970

[102] M. W. Wandling , A. B. Nathens , M. B. Shapiro , and E. R. Haut , “Police Transport Versus Ground EMS: A Trauma System‐Level Evaluation of Prehospital Care Policies and Their Effect on Clinical Outcomes,” Journal of Trauma and Acute Care Surgery 81, no. 5 (2016): 931–935, 10.1097/TA.0000000000001228. 27537514

[103] J. Glatts , J. Weissenburger , M. Mullen‐Fortino , L. Mazzone , and P. Z. Cacchione , “Patient Extrication Process for Urban Emergency Departments,” Journal of Emergency Nursing 48, no. 3 (2022): 328–338, 10.1016/j.jen.2022.01.012. 35526878

[104] R. A. Band , R. A. Salhi , D. N. Holena , E. Powell , C. C. Branas , and B. G. Carr , “Severity‐Adjusted Mortality in Trauma Patients Transported by Police,” Annals of Emergency Medicine 63, no. 5 (2014): 608–614.e3, 10.1016/j.annemergmed.2013.11.008. 24387925 PMC5912155

[105] A. Alshibani , M. Alharbi , and S. Conroy , “Under‐Triage of Older Trauma Patients in Prehospital Care: A Systematic Review,” European Geriatric Medicine 12, no. 5 (2021): 903–919, 10.1007/s41999-021-00512-5. 34110604 PMC8463357

[106] M. D. McPherson , M. Baxter , R. Crouch , and V. MacArthur , “The Process of Identifying Major Trauma in the Older Person in a Single Major Trauma Centre: A Service Evaluation,” International Emergency Nursing 69 (2023): 101283, 10.1016/j.ienj.2023.101283. 37257362

[107] R. V. Anantha , M. D. Painter , F. Diaz‐Garelli , et al., “Undertriage Despite Use of Geriatric‐Specific Trauma Team Activation Guidelines: Who Are We Missing?,” American Surgeon 87, no. 3 (2021): 419–426, 10.1177/0003134820951450. 33026234

[108] M. A. Horst , M. E. Morgan , T. M. Vernon , et al., “The Geriatric Trauma Patient: A Neglected Individual in a Mature Trauma System,” Journal of Trauma and Acute Care Surgery 89, no. 1 (2020): 192–198, 10.1097/TA.0000000000002646. 32118822

[109] G. Fuller , A. Pandor , M. Essat , et al., “Diagnostic Accuracy of Prehospital Triage Tools for Identifying Major Trauma in Elderly Injured Patients: A Systematic Review,” Journal of Trauma and Acute Care Surgery 90, no. 2 (2021): 403–412, 10.1097/TA.0000000000003039. 33502151

[110] L. M. Kodadek , S. Selvarajah , C. G. Velopulos , E. R. Haut , and A. H. Haider , “Undertriage of Older Trauma Patients: Is This a National Phenomenon?,” Journal of Surgical Research 199, no. 1 (2015): 220–229, 10.1016/j.jss.2015.05.017. 26070496

[111] J. M. Bardes , E. Benjamin , M. Schellenberg , K. Inaba , and D. Demetriades , “Old Age With a Traumatic Mechanism of Injury Should Be a Trauma Team Activation Criterion,” Journal of Emergency Medicine 57, no. 2 (2019): 151–155, 10.1016/j.jemermed.2019.04.003. 31078345

[112] J. A. Llompart‐Pou , J. Pérez‐Bárcena , M. Chico‐Fernández , M. Sánchez‐Casado , and J. M. Raurich , “Severe Trauma in the Geriatric Population,” World Journal of Critical Care Medicine 6, no. 2 (2017): 99–106, 10.5492/wjccm.v6.i2.99. 28529911 PMC5415855

[113] M. Fröhlich , M. Caspers , R. Lefering , et al., “Do Elderly Trauma Patients Receive the Required Treatment? Epidemiology and Outcome of Geriatric Trauma Patients Treated at Different Levels of Trauma Care,” European Journal of Trauma and Emergency Surgery 46, no. 6 (2020): 1463–1469, 10.1007/s00068-019-01285-0. 31844920

[114] J. B. Brown , M. L. Gestring , R. M. Forsythe , et al., “Systolic Blood Pressure Criteria in the National Trauma Triage Protocol for Geriatric Trauma: 110 Is the New 90,” Journal of Trauma and Acute Care Surgery 78, no. 2 (2015): 352–359, 10.1097/TA.0000000000000523. 25757122 PMC4620031

[115] T. Garwe , C. D. Newgard , K. Stewart , et al., “Enhancing Utility of Interfacility Triage Guidelines Using Machine Learning: Development of the Geriatric Interfacility Trauma Triage Score,” Journal of Trauma and Acute Care Surgery 94, no. 4 (2023): 546–553, 10.1097/TA.0000000000003846. 36404409 PMC10038832

[116] K. Russell and S. Biswas , “Pediatric Trauma Center vs. Adult Trauma Center: Which Is Better?,” Current Opinion in Anaesthesiology 36, no. 2 (2023): 159–162, 10.1097/ACO.0000000000001245. 36745064

[117] M. Podda , B. De Simone , M. Ceresoli , et al., “Follow‐up Strategies for Patients With Splenic Trauma Managed Non‐Operatively: The 2022 World Society of Emergency Surgery Consensus Document,” World Journal of Emergency Surgery 17, no. 1 (2022): 52, 10.1186/s13017-022-00457-5. 36224617 PMC9560023

[118] L. Moore , G. Freire , A. F. Turgeon , et al., “Pediatric vs Adult or Mixed Trauma Centers in Children Admitted to Hospitals Following Trauma: A Systematic Review and Meta‐Analysis,” JAMA Network Open 6, no. 9 (2023): e2334266, 10.1001/jamanetworkopen.2023.34266. 37721752 PMC10507486

